# Acceptability of digital health technologies in early Parkinson's disease: lessons from WATCH-PD

**DOI:** 10.3389/fdgth.2024.1435693

**Published:** 2024-08-26

**Authors:** T. Kangarloo, R. D. Latzman, J. L. Adams, R. Dorsey, M. Kostrzebski, J. Severson, D. Anderson, F. Horak, D. Stephenson, J. Cosman

**Affiliations:** ^1^ Takeda Pharmaceuticals, Cambridge, MA, United States; ^2^ Center for Health+Technology, University of Rochester, Rochester, NY, United States; ^3^ Department of Neurology, University of Rochester, Rochester, NY, United States; ^4^ Clinical Ink, Horsham, PA, United States; ^5^ Balance Disorders Laboratory, Department of Neurology, Oregon Health and Science University, Portland, OR, United States; ^6^ Critical Path Institute, Tuscan, AZ, United States; ^7^ Abbvie, North Chicago, IL, United States

**Keywords:** digital tool, patient feedback, Parkinson, wearability, wearable sensors

## Abstract

**Introduction:**

Digital health technologies (DHTs) have the potential to alleviate challenges experienced in clinical trials through more objective, naturalistic, and frequent assessments of functioning. However, implementation of DHTs come with their own challenges, including acceptability and ease of use for study participants. In addition to acceptability, it is also important to understand device proficiency in the general population and within patient populations who may be asked to use DHTs for extended periods of time. We thus aimed to provide an overview of participant feedback on acceptability of DHTs, including body-worn sensors used in the clinic and a mobile application used at-home, used throughout the duration of the Wearable Assessments in the Clinic and at Home in Parkinson's Disease (WATCH-PD) study, an observational, longitudinal study looking at disease progression in early Parkinson's Disease (PD).

**Methods:**

82 participants with PD and 50 control participants were enrolled at 17 sites throughout the United States and followed for 12 months. We assessed participants' general device proficiency at baseline, using the Mobile Device Proficiency Questionnaire (MDPQ). The mean MDPQ score at Baseline did not significantly differ between PD patients and healthy controls (20.6 [2.91] vs 21.5 [2.94], *p* = .10).

**Results:**

Questionnaire results demonstrated that participants had generally positive views on the comfort and use of the digital technologies throughout the duration of the study, regardless of group.

**Discussion:**

This is the first study to evaluate patient feedback and impressions of using technology in a longitudinal observational study in early Parkinson's Disease. Results demonstrate device proficiency and acceptability of various DHTs in people with Parkinson's does not differ from that of neurologically healthy older adults, and, overall, participants had a favorable view of the DHTs deployed in the WATCH-PD study.

## 1 Introduction

Advances in digital technologies, such as mobile phones and wearables, are now ubiquitous and have changed how we interact with others and the world around us. For example, a 2020 poll showed that 90% of Americans own a smartphone and 21% own a smartwatch or fitness tracker (1, 2). Beyond giving us the capabilities to post pictures, play games, or track our workouts, these technologies have become particularly valuable in the health and research sectors (3). In clinical trials, for example, as opposed to traditional assessments, which are subjective and performed infrequently, digital tools have the potential to provide a more holistic view of disease symptoms (4–6), progression (7–9), and response to treatment (5). Furthermore, using digital tools in fully decentralized or hybrid clinical trials can reduce or fully eliminate site visits, a documented barrier to clinical trial participation due to patient and caregiver burden (10).

Although using digital health technologies (DHTs) may alleviate some of the challenges faced in clinical trials, they often come with their own challenges resulting in lower rates of adaptation, particularly among older individuals. There is a false assumption of device proficiency in the general population, especially when working with a population of older adults, who require greater assistance in relation to digital technologies than younger populations (11). For instance, a nonexperimental study design exploring attitudes about technology in older adults found that older adults were willing to use technology but had negative outlooks associated with technology creating inconveniences and unhelpful features, thus making it harder to use and navigate (12). Other factors that have contributed to low technology adaptation in older adults include poor technology designs that don't consider the perceptual and cognitive abilities of older adults, and poor training on use of the technology (13).

One disease consisting primarily of older adults where the use of DHTs has been especially relevant in clinical trial measurement is Parkinson's Disease (PD). PD, the second most prevalent and fastest growing movement disorder in the world, affects about 1% of adults 60 years and older (14). The cardinal features of the disease are motor impairments such as tremor, rigidity, and bradykinesia, however, the clinical features extend beyond that as patients typically bring to light the cognitive and mood impairments caused by the disease (15, 16). The current gold standard for assessing progression in PD, the Movement Disorders Society Unified Parkinson's Disease Rating Scale (MDS-UPDRS) (17), has limitations which pose challenges for clinical trials. For instance, to properly power a phase II clinical trial to see a change in the MDS-UPDRS studies must have large sample sizes and long study durations (18, 19). The frequency in which participants need to come into the clinic in traditional clinical trials can also be a hurdle as clinical trials are typically run in large, academic hospitals researchers are only capturing participants that live in metropolitan areas or have the means to travel to study sites (20). Using digital technologies in clinical trials can not only give us better, more sensitive, measures of disease progression but can also help us reach a wider range of participants by reducing the number of clinic visits or potentially shifting towards totally remote clinical trials.

One method to assess comfort with technology in older adults is the Mobile Device Proficiency Questionnaire (MDPQ). The MDPQ includes items related to comfort using devices, such as tablets and smartphones, and has been found to be a highly reliable measure of mobile device proficiency in older adults (21). The MDPQ could serve as a tool to identify participants who may need more training in using digital technologies in clinical trials. Additionally, researchers can evaluate patients’ first-hand experiences using DHTs by harnessing the voice of the individuals participating in research studies and clinical trials. Acquiring patient feedback early and often, through panels, interviews, and questionnaires, can provide insights related to the acceptability of these technologies and help inform future study design.

### 1.1 Current study

The Wearable Assessments in the Clinic and at Home in Parkinson's Disease [WATCH-PD (4);] study was a one-year, observational study exploring disease progression using DHTs in early Parkinson's Disease. Perceptions of the DHTs used in the WATCH-PD study were captured from participants throughout the study. In this paper we aim to give an overview of participant feedback with the goal of providing a better understanding of the feasibility and burden of using these technologies during participation in longitudinal clinical trials. Specifically, we aimed to report if there are differences between people with PD and control participants in (1) device proficiency at baseline as measured by the MDPQ and (2) overall impressions of using digital technologies during participation in a 12-month longitudinal study.

## 2 Methods

### 2.1 Trial design

The Wearable Assessments in the Clinic and at Home in PD is a prospective, longitudinal, multisite natural history study in people with early, untreated PD (<2 yr since diagnosis) and neurologically healthy matched controls. 82 participants with PD and 50 control participants were enrolled at 17 sites throughout the United States and followed for 12 months. Participants completed regular clinic visits in addition to completing self-administered assessments of motor and non-motor function outside of the clinic using a mobile application twice monthly. A brief description is provided below. For a fuller description, please see Adams et al. (4).

### 2.2 Participants

Participants were recruited from clinics, study interest registries, and social media. We aimed to evaluate a population similar to the Parkinson's Progression Markers Initiative (PPMI) (22). Thus, at enrollment, PD patients were required to be aged 30 or older, within 2 years of diagnosis, untreated with symptomatic medications [including levodopa, dopamine agonists, Monoamine oxidase-B (MAO-B) inhibitors, amantadine, anticholinergics] and not expected to require medication for at least 6 months at baseline, a modified Hoehn and Yahr ≤2, and at least two of the following symptoms: resting tremor, bradykinesia, or rigidity (must have either resting tremor or bradykinesia as one of two symptoms); OR either asymmetric resting tremor or asymmetric bradykinesia. Control participants were required to be aged 30 or older at the time of enrollment, with no diagnosis of a significant Central Nervous System (CNS) disease (other than PD), history of repeated head injury, history of epilepsy or seizure disorder other than febrile seizures as a child, or history of a brain magnetic resonance imaging (MRI) scan indicative of clinically significant abnormality. For both PD patients and controls, a Montreal Cognitive Assessment (MoCA) score < 24 was considered exclusionary.

### 2.3 Study assessments

Each participant completed clinic visits at Screening/Baseline, 1, 3, 6, 9, 12 months. Clinic visits consisted of three core components: (1) a comprehensive battery of clinician and patient-reported outcomes measuring both motor and non-motor function, (2) a set of motor assessments completed while wearing inertial sensors distributed across the body, and (3) completion of a custom-developed, self-administered mobile phone battery designed to measure aspects of motor and non-motor function. In addition to in-clinic assessments, participants were asked to wear a smartwatch on the wrist of their most affected side for 7 days following each clinic visit and were asked to complete the same mobile battery they completed during clinic visits every two weeks for the duration of the study. Due to COVID-19 a subset of individuals did some of the in-clinic assessments remotely and not all data were available.

### 2.4 Instrumented motor assessments

**Figure 1:**
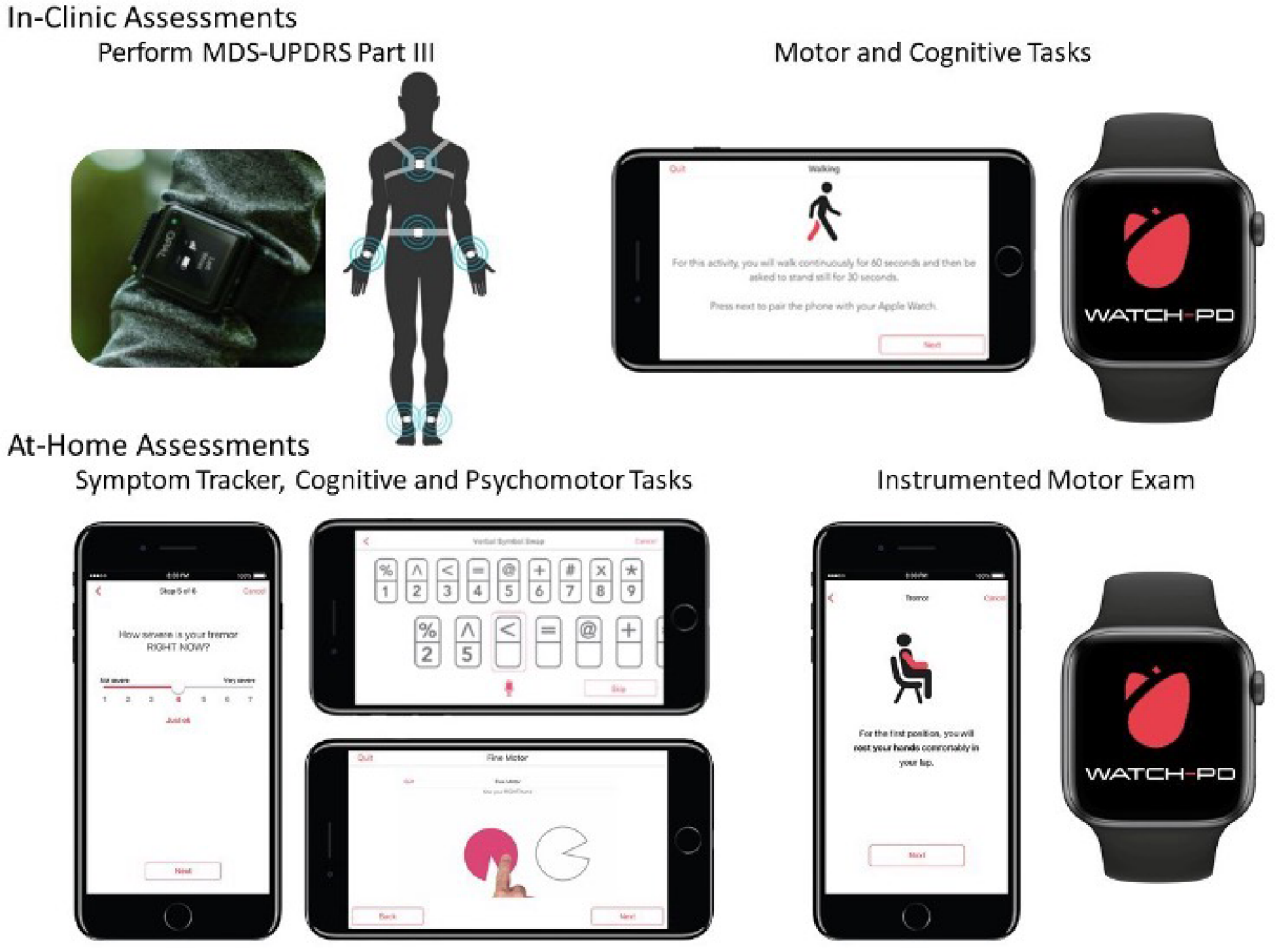
Digital devices evaluated in-clinic and at-home during WATCH-PD. Adapted from Adams et al. (4).

At each clinic visit, participants were instrumented with a set of six Opal sensors (OPAL system, APDM, Inc., Portland, OR, United States) placed on each wrist, around each foot, and one sensor each positioned on the sternum and the lumbar area (Figure 1). The Opal sensors contain 3-axis, accelerometers, gyroscopes, and magnetometers, and were used to capture raw kinematic data during the performance of the MDS-UPDRS Part III motor examination, as well as a 5× sit-to-stand task, a 30 s standing balance task (eyes open), a two-minute walking task and a two-minute walking task under cognitive load (serial sevens).

### 2.5 Mobile assessment battery

As noted above, participants were provided with a provisioned smartphone and smartwatch and completed a custom-designed mobile battery, developed by Clinical Ink (Clinical Ink, Horsham, PA USA), at each clinic visit and every two weeks during their participation in the study. The purpose of completing the mobile battery during clinic visits was twofold. First, it provided a means of orienting participants to the devices and tasks to be performed. In addition, it allowed comparison of performance measures derived from the mobile assessments to contemporaneously collected measures acquired through the Opal system and clinician and patient reported outcomes completed during each visit. The mobile battery took approximately 15–20 min to complete, and participants were asked to complete the entire battery at once. They were allowed up to an hour to complete the tasks, providing time for unexpected interruptions or breaks. The battery consisted of three core components, measuring both motor and non-motor domains. First, participants completed a set of six, brief PRO questions, providing responses on a 1–7 Likert scale with questions related to current mood, fatigue, sleepiness, and cognition, as well as the current severity of bradykinesia and tremor (Table 1). Participants then completed a set of brief cognitive and psychomotor tasks and a brief speech recording battery. Finally, participants completed a brief instrumented motor exam consisting a of a 1-minute walking task, a 30 s balance task, and 20 s resting and postural tremor tasks.

**Table 1:** Results of the mobile device proficiency questionnaire (MDPQ) in Parkinson's disease participants and controls at baseline.

	PD (*N* = 82)	Control (*N* = 50)	*P*-value
MDPQ score			
Mean (SD)	20.6 (2.91)	21.5 (2.94)	0.0962
Median [Min, Max]	20.0 [15.0, 25.0]	20.5 [15.0, 25.0]	

### 2.6 Mobile device proficiency questionnaire

At baseline participants completed an abridged version of the MDPQ focused on a subscale of Mobile Device Basics most relevant to the tools being used in the current study. The MDPQ Mobile Device Basics subscale is comprised of nine questions that ask participants to rate their ability to perform tasks on a smartphone or tablet device on a 1–5 Likert scale (1 = never tried, 2 = not at all, 3 = not very easily, 4 = somewhat easily, 5 = very easily). The MDPQ was available for all participants at Baseline.

### 2.7 Wearability and comfort questionnaire

At baseline, Months 1, 6, and end of study (month 12), participants were asked to take a questionnaire with quantitative questions related to using the digital technologies both in the clinic and at home (Supplemental 1). Quantitative questions relating to comfort, ease of use, and burden were either on a Likert or categorical (Yes/No/Neutral) scale. The Likert Scale was a 1 to 5 scale for both comfort of devices (1 = Very Acceptable, 2 = Acceptable, 3 = Neutral, 4 = Unacceptable, 5 = Very Unacceptable) and ease of use (1 = Very easy, 2 = Easy, 3 = Neutral, 4 = Difficult, 5 = Very Difficult). At the end of the study, participants completed an exit questionnaire which addressed qualitative questions related to the use of the devices, and non-device questions related to length of study and compensation.

At baseline, the Wearability and Comfort Questionnaire was available for 80 participants with PD and 49 controls, however some questions were left blank which is reflected in our results. At month 1, the Wearability and Comfort Questionnaire was available for 72 participants with PD and 40 controls and at month 12, it available for 80 participants with PD and 46 controls.

### 2.8 Statistical analysis

Descriptive statistics for the MDPQ total subset score and Wearability and Comfort Questionnaire scores at Baseline, Month 1, and Month 12 were reported for PD participants and controls. A two-tailed *t*-test was performed between PD participants and controls on the MDPQ to determine if there was a difference in scores between the two groups where *p* < 0.05 was considered statistically significant. All analyses were performed in R Statistical Software (v4.1.2; R Core Team 2021).

## 3 Results

### 3.1 Mobile device proficiency questionnaire

Table 2 summarizes the results of the MDPQ at Baseline. The mean [SD] score in PD participants [20.6 (2.91)] was numerically smaller than controls [21.5 (2.94)] but did not differ significantly across the two groups (*p* = 0.10).

**Table 2:** Description and location of assessments conducted with the digital devices used in WATCH-PD^a^.

Device	Assessment	Description	Location
Wearable sensors	Timed walk test	The participant is timed while walking for a distance of 10 meters. The individual walks the 10-m path back and forth, turning at the end of their path, for 2 min.	In-clinic
	Repeat timed walk test with serial sevens	The participant repeats the Timed Walk Test described above. While walking, the participant performs a serial subtraction of sevens beginning with the number 100.	
	Sit-to-stand test	The participant sits against the back of a chair and stands up as quickly as they can for 5 repetitions without stopping.	
	Postural sway	The participant stands still, looking straight ahead for 30 s.	
Smartphone application	Symptom tracker	The participant answers a 5-item survey on the phone including questions about mood, sleepiness, thinking, tremor severity, and difficulty with movement.	In-clinic and remotely biweekly
	Symbol digit modalities test	The participant is given a key that connects symbols to numbers. The participant is presented with a symbol and must speak aloud the corresponding number.	
	Trail making test	The participant must connect a set of dots as quickly as possible using the index finger on their dominant hand while still maintaining accuracy.	
	Visuospatial working memory	The participant is briefly shown four colored boxes. The participant is then shown a single-colored box and must indicate if that box was in the previous set of four.	
	Finger tapping	The participant performs rapid alternating finger movements by tapping two targets that appear side by side using their index and middle fingers.	
	Fine motor test	The participant is presented with a pink object and an outline. The individual must use 1–2 fingers to move and rotate the object into the outline as quickly as possible.	
	Speech assessment	Participants must perform a sustained phonation task, a verbal articulation task repeating the syllables “pa ta ka,” and a sentence reading task.	
Smartwatch	Timed walk test	The participant must walk in a straight line, turning at the end of their path, for 1 min.	In-clinic and remotely biweekly
	Balance test	The participant must stand still with their arms at their side for 30 s.	
	Tremor task	The participant must rest their hands in their lap for 10 s, then extend their arms out in front of them for 10 s.	

^a^ Table 2 adapted from Adams et al. (4).

### 3.2 Wearability and comfort questionnaire

**Figure 2:**
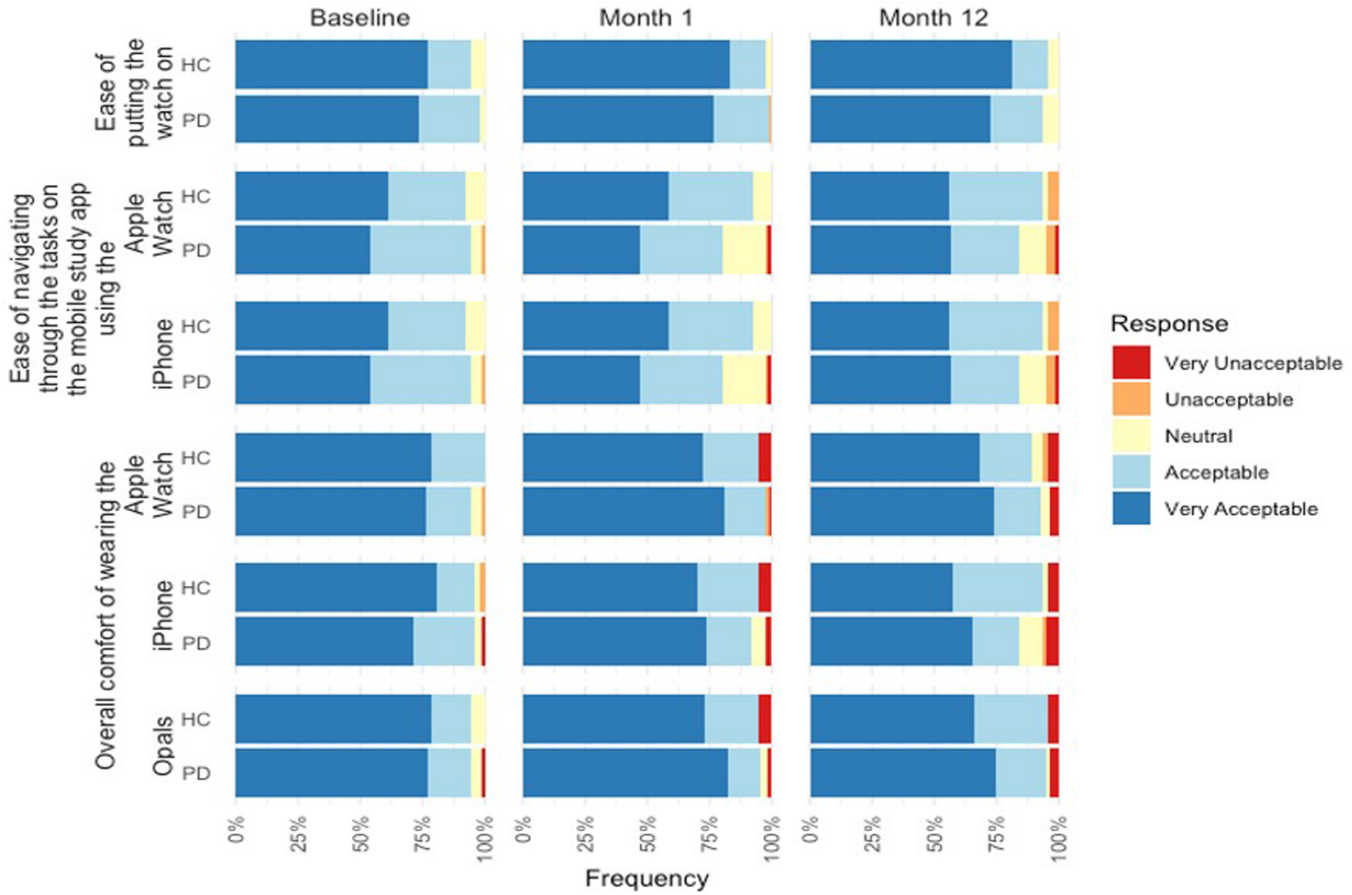
Impressions of using DHTs at baseline, Months 1, and 12 of PD and control participants.

Figure 2 summarizes the results of the Wearability and Comfort Questionnaire at Baseline, Month 1, and Month 12.

### 3.3 Baseline

For overall comfort of the devices, the majority of the PD participants (75.9%) found the comfort of wearing the Opals to be very acceptable. Positive feedback was also reported for the mobile phone and smartwatch with the majority of participants reporting the comfort of the devices very acceptable (71.2% and 78.8% respectively). Similarly, controls reported very acceptable comfort for the Opals (77.6%), mobile phone (79.6%), and smartwatch (77.6%).

**Figure 3:**
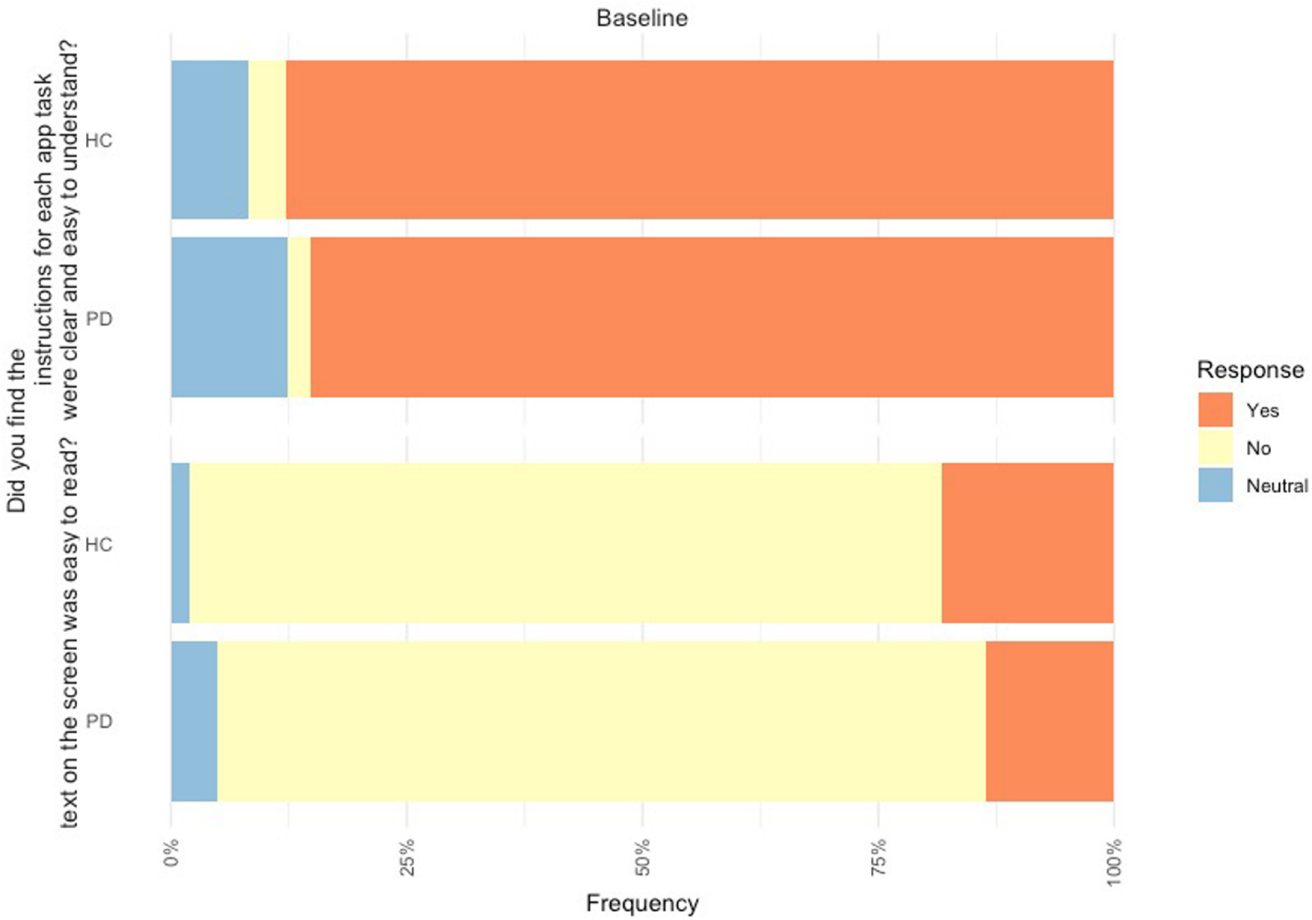
Feedback of mobile device platform at baseline from PD participants (n = 80) and controls (n = 49).

In relation to the mobile assessment, 85.2% of PD participants found the instructions on the mobile assessment to be clear and easy to understand, but 81.5% found the text was not easy to read. Likewise, 87.8% of controls found the instructions on the mobile assessment easy to be clear and easy to understand and 79.6% found the text was not easy to read (Figure 3).

### 3.4 Month 1

For overall comfort of the devices in Month 1, the majority of the PD participants (82.1%) found the comfort of wearing the Opals to be very acceptable. Positive feedback was also reported for the mobile phone and smartwatch with the majority of participants reporting the comfort of the devices very acceptable (74.0% and 80.8% respectively), including acceptability of putting the smartwatch on at home (76.7%). Similarly, controls reported very acceptable comfort for the Opals (73.0%), mobile phone (70.0%), and smartwatch (72.5%), and 83.0% reported the ease of putting on the smartwatch as very acceptable.

### 3.5 Month 12

For overall comfort of the devices in Month 12, the majority of PD participants (74.4%) found the comfort of wearing the Opals to be very acceptable. Positive feedback was also reported for the mobile phone and smartwatch with the majority of participants reporting the comfort of the devices very acceptable (65.0% and 73.8% respectively), including acceptability of putting the smartwatch on at home (72.5%). Similarly, controls reported very acceptable comfort for the Opals (66.0%), mobile phone (57.4%), and smartwatch (68.1%), and 83.0% reported the ease of putting on the smartwatch as very acceptable.

Highlights of the qualitative feedback related to the devices at Month 12 was grouped and can be found in Table 3. Participants highlighted the need for a better watch strap, more notifications on the mobile device to complete the battery, and frustrations with technological issues.

**Table 3:** Qualitative feedback from participants on use of smartwatch and smartphone at-home in WATCH-PD.

Smartwatch	Smartphone
*“Too bulky”* *“Sometimes when tremors are acting up, the watch was uncomfortable”* *“The strap interfered with writing and using a computer mouse”* *“Would prefer to wear it on non-dominant wrist”* *“The walking activity, when both the phone and watch are used alternatively, was confusing”* *“Would be helpful to have study reminders on the watch”*	*“The study phone was just a brick when not used for sessions”* *“Sometimes between uses the battery dies so a call or text would be helpful”* *“A call or text on my personal phone to remind me about study tasks would be helpful”* *“Froze once so I had to reboot”*

## 4 Discussion

This work aimed to gather participant perceptions of the DHTs used in the WATCH-PD study. This is the first study to evaluate feedback and impressions of using common DHTs in both controls and people with early PD in the context of a longitudinal, observational study. We show that for an early PD population, experiences and comfort with technology are not different from the general experience in neurologically healthy older adults. Furthermore, there was an overall favorable view of the usability and comfort of the digital technologies deployed in the WATCH-PD study, both in-clinic and at-home.

Results from the MDPQ mobile subscale at baseline demonstrated no significant differences in device proficiency between the PD participants and controls. The results from the Wearability and Comfort Questionnaire overall demonstrated generally positive views on the comfort and use of the digital technologies in this study. Consistently, over the 12-month study duration, within both cohorts, most participants found wearing the Opal sensors, mobile phone, and smartwatch either very acceptable or acceptable regarding comfort. The ease of putting on the Apple Watch band was also favorable throughout the study, which was encouraging given that many of the PD participants presented with tremor dominant symptoms at baseline.

The study is not without limitations. The baseline MDPQ scores combined with the highly positive results on the Wearability and Comfort Questionnaire might suggest that the study was biased towards recruiting people who were already very comfortable with technology. This cohort was also homogenous, potentially limiting the generalizability of our findings. Thus, it is recommended that future work collect similar measures in more diverse cohorts, potentially through a fully remote study design to widen recruitment and include a broader range of individuals. Moreover, there were a few limitations which we could not control, including the maximum size of the screen of the mobile device.

## 5 Conclusions

The current research in early PD, along with extant literature on DHT usability and acceptability more generally, provides a foundation for understanding the acceptability of using digital tools in early PD clinical trials. Our work provides insights into how older individuals, especially those with a movement disorder, will adapt to using digital technologies in clinical trials. A key to overcoming possible challenges with the use of DHTs in older participants with neurological disorders is to incorporate the patient voice by gathering regular formal and informal feedback throughout study design and conduct. Furthermore, the option of co-design with the end users provides an opportunity to collect valuable feedback and create a collaborative experience between researchers and patients.

## Data Availability

The datasets presented in this article are not readily available because data is available to members of the Critical Path for Parkinson's Consortium 3DT Initiative Stage 2. For those who are not a part of 3DT Stage 2, a proposal may be made to the WATCH-PD Steering Committee (via the corresponding author) for de-identified datasets. Requests to access the datasets should be directed to Jamie.Adams@chet.rochester.edu.
